# Oil as a Hindrance to Oat (*Avena sativa* L.) Nutrient Fractionation: Leveraging Mass Spectrometry-Based Omics to Unravel Lipid Regulation for Functional Crop Improvement

**DOI:** 10.3390/foods15071224

**Published:** 2026-04-03

**Authors:** Darren Lau, Leigh Donnellan, John C. Harris, Peter Hoffmann

**Affiliations:** 1 School of Pharmacy and Biomedical Science, College of Health, Adelaide University, Adelaide 5000, Australia; darren.lau@adelaide.edu.au (D.L.); leigh.donnellan@adelaide.edu.au (L.D.); 2 South Australian Research and Development Institute, Department of Primary Industries and Regions, Adelaide 5000, Australia; john.harris2@sa.gov.au

**Keywords:** oat (*Avena sativa* L.), processing and extraction, oil synthesis, proteomics, lipidomics

## Abstract

Oats (*Avena sativa* L.) are a nutritionally valuable cereal crop known for their unique profile of bioactive compounds, including protein, β-glucan (BG), and avenanthramides (AVNs). However, industrial-scale processing and fractionation of these nutrients at an industrial scale are restricted by high oil content, limiting their application as functional food ingredients. While reducing oil content through targeted breeding may overcome these barriers, this strategy requires a deeper molecular understanding of lipid metabolism and its interplay with other nutrient pathways. In this review, we highlight the health benefits of key oat nutrients and discuss challenges in isolation techniques at an industrial scale. We then outline the canonical pathway for seed oil biosynthesis, supported by functional validation of genes encoding key lipid synthesis enzymes, and review studies linking regulatory enzymes to variations in oat oil content at gene and transcript levels. Finally, we highlight how mass spectrometry-based omics, particularly proteomics and lipidomics, can be used in breeding programmes to elucidate regulatory networks involved in oat oil biosynthesis and nutrient partitioning at the phenotype level.

## 1. Introduction

Oat (*Avena sativa* L.) is an important cereal grain crop, having been cultivated for over 2000 years. In 2022 alone, more than 26 million tonnes of oats were produced, with Canada, and Australia being the largest producers. However, this figure still places oat at seventh place, in terms of global production quantity, among all cereal grains (https://www.fao.org/faostat/en/, accessed 31 December 2025). Oat production has been facing a declining trend over the past 40 years. This is partly due to its plantation mainly catering for the livestock industry; oats are often priced through feed market mechanisms, leading farmers to opt for more profitable crops.

Oats have a nutrition profile with significant health benefits that are often overlooked. Oats contain higher levels of protein with lower allergenicity than other cereal counterparts, soluble fibre such as β-glucan (BG), and unique phytochemicals such as avenanthramides [1,2,3]. With increased public health consciousness and shifting dietary preferences towards more sustainable protein sources, oat presents as an ideal plant-based alternative in the market [4]. Diversification of oats’ role as a functional ingredient can support consumers’ growing interest in this cereal crop, ultimately increasing its value at the farm gate.

The isolation of protein and BG concentrates from milled oats is often performed through dry separation techniques at an industrial level. However, high oil levels in oats, another unique characteristic of this cereal crop, contribute to aggregate formation during these steps, hindering processing efficiency. These challenges can be addressed by removing oil from partially milled oats using supercritical carbon dioxide (SC-CO_2_) extraction [5]; however, this process is cost-intensive. Genetic manipulation for low-oil oats can be more cost-effective, but requires a deeper understanding of oil synthesis regulation in oats and its impact on further grain quality characteristics.

“Omics” technologies offer a comprehensive approach to analyse a suite of biological molecules in one analysis, including genes (genomics), mRNA (transcriptomics), proteins (proteomics), and metabolites or lipids (metabolomics or lipidomics) [6]. Genomics and transcriptomics are popular approaches used by agricultural scientists to understand how alleles contribute to variation in crop quality traits [7]. However, gene-level information does not always translate to the plant phenotype. On the other hand, proteomics and metabolomics or lipidomics offer direct insights into a plant’s physiological state. As proteins and metabolites are functional products of gene expression, proteomics and metabolomics reflect real-world phenotypic outcomes [8,9], circumventing or unveiling levels of pre- and post-translational regulatory mechanisms. Through the global profiling of molecular regulators, we may identify key targets to reduce oil content and understand how this may affect carbon partitioning into major nutrients such as starch and protein, and also BG.

In this review, we describe the health benefits of the different oat nutrient fractions, particularly protein, BG, and oil. The associated challenges with isolation steps of these fractions at laboratory and industrial scales are also discussed. Addressing the primary issue of oat processing and fractionation, we review the canonical oil synthesis pathways in plants and how this can be studied in oat via mass spectrometry-based omics approaches in efforts to explore the molecular regulation of oat oil synthesis, accumulation, and nutrient partitioning at the phenotype level.

## 2. Oil as a Processing Barrier to Oat Nutrient Fractionation

### 2.1. Oat Nutrient Profile

Oats have a distinct nutrient profile, as characterised by their high oil, protein, and dietary fibre content, as well as the low molecular weight bioactive phenolic compounds AVNs that are unique to oats. The increasing evidence supporting the health benefits of oat consumption has driven interest from the food industry to incorporate oats as ingredients for new product development.

A distinct characteristic of oat grains is their high oil content, which can achieve up to 20% in some varieties [10,11]. Triacylglycerol (TG) is the major lipid class in oat oil, constituting up to around 80% of total lipids, followed by phospholipids (6 to 26%), such as phosphatidylcholine (PC) [11]. Oat oil has been a popular product in the cosmetic industry due to the presence of ceramides, which are known to have skin-protective properties [12]. However, its feasibility for dietary purposes remains limited. Oat oil also demonstrated cholesterol-lowering effects by promoting faecal lipid and bile acid excretions when fed to rats [13]. The use of ethanol as a co-solvent during SC-CO_2_ extraction of oat oil may also extract other polyphenols, hence improving the nutritional quality of the product [14,15]. A recent study has also suggested the utilisation of oat oil for microencapsulation applications. The presence of oat oil was suggested to enhance the nutritional profile of the emulsion system and improve the body’s ability to absorb the encapsulated green tea polyphenols [16].

Oats are considered a high-protein cereal, with content ranging from 10 to 18%, and are a good source of bioactive peptides [17,18,19]. Due to their avenalin-rich composition, unlike wheat and barley that are mainly made up of prolamins, oat proteins are usually considered tolerable by patients with coeliac disease. Avenalins are the major storage proteins in oats, accounting for up to 80% of the total oat protein. A secondary protein, called avenin, is more similar to wheat and barley prolamins or gluten proteins [20]. However, the mapping of coeliac-associated T-cell epitopes to predicted avenin proteins showed very low prevalence of coeliac-associated immune-reactive regions [21]. This supports the suitability of oats in gluten-free diets, providing a source of whole-grain fibre.

Oats are also rich sources of soluble fibres, particularly mixed linkage (1–3,1–4)-β-D-glucan, also commonly known as BG. BG content in oats ranges from 3 to 7% by weight [22,23]. After consumption, BGs are fermented by gut bacteria to produce short-chain fatty acids with roles in maintaining a healthy gut microbiome [24,25,26,27]. By forming a viscous substance in the small intestine, BGs decrease the rate of cholesterol and bile acid absorption into the bloodstream, leading to excretion from the body [28]. As bile acid absorption is inhibited, circulating LDL cholesterol is redirected towards bile acid reproduction, thus reducing its levels in the blood [29]. Moreover, BG’s contribution to blood sugar control in type-2 diabetic patients is well-studied [30,31,32]. BG consumption leads to a gradual decrease in blood sugar levels by reducing the absorption rate of carbohydrates, improving insulin responses, and reducing the risk of hyperglycaemia [33,34,35,36].

### 2.2. Extraction of Oat Nutrients at Industrial Scale

Recognising the nutritional value of oat bran highlights the importance of efficiently utilising oats to develop value-added products. Protein and β-glucan fractions can be enriched from milled oats using wet or dry separation techniques. While wet extraction methods are effective in producing high-purity isolates, they are associated with high production costs due to solvent use and downstream drying requirements. In contrast, dry separation techniques are more cost-effective and better preserve the structural and physicochemical properties of nutrients, although enrichment efficiency is generally lower. These methods typically involve milling oat grains into smaller particles, followed by separation based on size or density using sieving or air classification, respectively [37].

**Figure 1:**
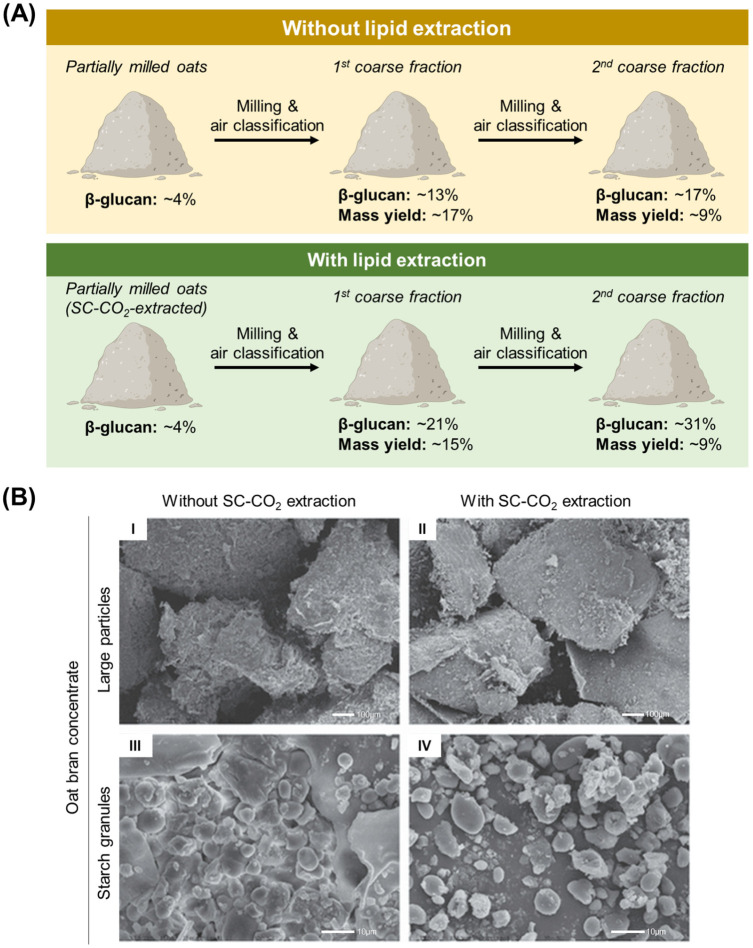
Lipid removal improves nutrient enrichment in oat fractions. (**A**) Approximate concentrations of β-glucan recovered without and with defatted oat flour following a two-step milling and fractionation process at pilot scale performed by Sibakov and colleagues [5]. Exact measurements with associated error ranges are presented in the original study. (**B**) Scanning electron micrographs of oat bran concentrate without (**I**,**III**) and with SC-CO_2_ treatment (**II**,**IV**). The exterior of oat bran particles appeared smoother, and starch granules were less aggregated after SC-CO_2_ lipid extraction. Adapted (from Figure 1) from Stevenson et al. [39] with permission from John Wiley and Sons.

As mentioned earlier, oats contain a higher percentage of oil and fat, as compared to other cereal grains. This characteristic presents a significant hurdle during the dry processing of oats, as it contributes to the clogging of machinery and ultimately lowers processing efficiency [5,38]. Defatting is therefore an essential step to improve enrichment of fractions with greater nutrient purity. A combination of further milling and air classification steps following SC-CO_2_ defatting of partially milled oat flakes was described by Sibakov et al., which resulted in finer flour in subsequent milling steps and enhanced separation of different oat fractions [5]. On a pilot scale, a fraction with ~31% β-glucan concentration (mass yield of ~9%) was achieved in defatted oats, as compared to ~17% (mass yield of ~9%) from non-defatted flour (Figure 1). Similar results were observed when performed on an industrial scale. Notably, a protein concentrate of 73.0% purity (mass yield of 5.0%) was achieved [5].

Several plausible mechanisms explain these observations. High oil content in oats may enhance the hydrophobicity of particle surfaces, which increases attractive interactions between starch granules. Larger particle sizes can also contribute to a higher magnitude of van der Waals attractive forces, further contributing to clumping and aggregation [40]. Lowering oil content may weaken the aggregation of starch granules, facilitating particle separation. A previous study also suggested that by reducing oat bran particle size using freeze-milling, lipid extraction can be improved and potentially enable greater yield of β-glucan recovery [41]. Additionally, SC-CO_2_ treatment has been shown to produce particles with smoother particle exterior and reduced starch granule aggregation (Figure 1B), thus further enhancing separation efficiency [39]. Ideally, the SC-CO_2_ extract, which consists of oat oil (and potentially bioactive compounds such as AVNs and phenolic acids if extracted in the presence of an organic co-solvent), can also be marketed as a valuable by-product. While performing SC-CO_2_ defatting of oats prior to milling may help to overcome this processing barrier, its broader implementation is constrained by high capital investment, elevated operating pressures, and increased energy demand, which may impact industrial scalability.

As an alternative approach, genetic manipulation could offer a promising solution. By selectively breeding or genetically engineering oats with lower oil content, the food industry may mitigate the need for extensive lipid extraction processes. However, this approach would require more research to thoroughly understand how oil biosynthesis is regulated in oats and the impact on other grain quality characteristics.

## 3. Regulation of Oat Oil Synthesis

### 3.1. Molecular Overview of Seed Oil Synthesis

**Figure 2:**
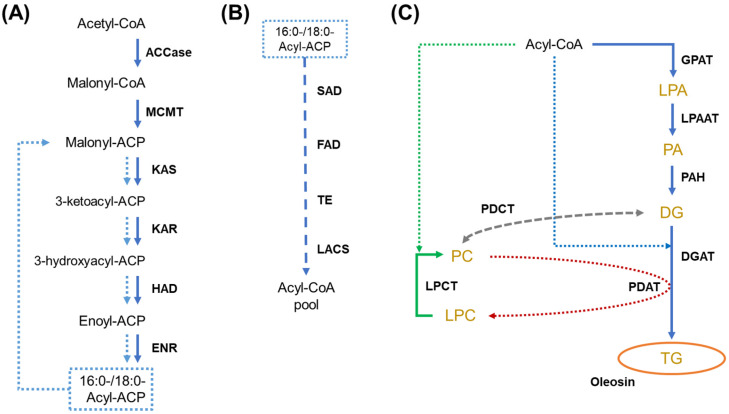
Brief overview of the canonical lipid synthesis pathway in seed crops. (**A**) Conversion of acetyl-CoA into saturated acyl-ACP (C16:0 and C18:0). (**B**) Further modification of acyl-ACP into acyl-CoA. (**C**) Assembly of acyl-CoA catalysed by multiple acyltransferases to form TG, the main lipid class of oat oil. Enzymes are represented in bold black font, while lipid classes are highlighted in gold font. Blue arrows indicate the de novo TG synthesis pathway. Green arrows highlight the conversion of LPC to PC, while brown arrow indicates conversion of PC to LPC. ACCase, acetyl-CoA carboxylase; ACP, acyl carrier protein; DG, diacylglycerol; DGAT, acyl-CoA:diacylglycerol acyltransferase; ENR, enoyl-CoA reductase; FAD, fatty acyl desaturase; GPAT, glycerol-3-phosphate acyltransferase; HAD, 3-hydroxyacyl-ACP dehydratase; KAR, 3-ketoacyl-ACP reductase; KAS, ketoacyl-ACP synthase; LACS, long-chain acyl-CoA synthetase; LPA, lysophosphatidic acid; LPAAT, lysophosphatidic acid acyltransferase; LPC, lysophosphatidylcholine; LPCT, lysophosphatidylcholine acyltransferase; MCMT, malonyl-CoA:ACP transacylase; PA, phosphatidic acid; PAH, phosphatidic acid phosphatase; PC, phosphatidylcholine; PDAT, phospholipid:diacylglycerol acyltransferase; PDCT, phosphatidylcholine:diacylglycerol cholinephosphotransferase; SAD, stearoyl-ACP desaturase; TG, triacylglycerol; TE, fatty acid thioesterase.

Fatty acid synthesis (FAS) and triacylglycerol assembly (TG) are two major pathways involved in seed oil synthesis. As highlighted in Figure 2A, acetyl-CoA molecules are first converted into saturated acyl-ACP (C16:0 and C18:0) molecules via the enzymatic activities of acetyl-CoA carboxylase (ACCase), malonyl-CoA:ACP transacylase (MCMT), 3-ketoacyl-ACP synthase (KAS), 3-ketoacyl-ACP reductase (KAR), 3-hydroxyacyl-ACP dehydratase (HAD), and enoyl-CoA reductase (ENR) [42]. Acyl-ACPs may undergo desaturation via stearoyl-ACP desaturase (SAD) and fatty acyl desaturase (FAD) to form unsaturated acyl-ACP molecules (C18:*n*-ACP; 0 < *n* ≤ 3), before being further modified by fatty acid thioesterase (TE) and long-chain acyl-CoA synthetase (LACS) into acyl-CoA esters (Figure 2B). As activated forms of fatty acids, acyl-CoAs act as precursors for TG synthesis via two main pathways. The de novo pathway, also known as the Kennedy pathway, involves the activities of glycerol-3-phosphate acyltransferase (GPAT), lysophosphatidic acid acyltransferase (LPAAT), phosphatidic acid phosphatase (PAH), and acyl-CoA:diacylglycerol acyltransferase (DGAT). This pathway converts acyl-CoA and glycerol-3-phosphate (G3P) into different lipid classes, including lysophosphatidic acid (LPA), phosphatidic acid (PA), diacylglycerol (DG), and ultimately TG [43]. DG can also give rise to TG formation through the flux of fatty acids from PC, involving different enzymatic mechanisms. Firstly, lysophosphatidylcholine (LPC) can contribute to the formation of PC through acyl editing by lysophosphatidylcholine acyltransferase (LPCT) (green arrows; Figure 2C). PC can then donate its acyl chain directly to DG and produce TG via phospholipid:diacylglycerol acyltransferase (PDAT) (dark red dashed arrow; Figure 2C). Lipid remodelling may also occur through the activity of phosphatidylcholine:diacylglycerol cholinephosphotransferase (PDCT), which governs the interconversion between PC and DG (grey dashed arrow; Figure 2C). TG molecules are then bound by oleosin proteins to form oil bodies as energy reserves [44].

Functional validation studies in various plant models have confirmed the involvement of several enzymes in FAS and TG assembly (Table 1). While the core FAS and TG assembly pathways are largely conserved across plant species, the regulation of these processes can vary substantially between crops. To date, there are very limited functional validation studies in oats for oat oil modulation. The recent literature using transgenic approaches demonstrated that the expression of oat *WRINKLED1* (*WRI1*) transcriptional factor in wheat endosperm increased oil content by almost 10-fold [45]. However, in transgenic oat lines, the overexpression of *WRI1*, along with *DGAT1* and *OLEOSIN*, only resulted in insignificant increases in seed oil content, suggesting the involvement of other oil synthesis regulators [46]. These findings highlight the need to identify further targets involved in oat oil biosynthesis.

**Table 1:** List of studies that demonstrated key roles of proteins in regulating seed oil synthesis using reverse genetic approaches.

Target	Organism	Method	Outcome	Ref.
*ACCase*	*Arabidopsis thaliana* *Camelina sativa*	Overexpression (OE) of *Pisum sativum α-carboxyltransferase* (ACCase subunit)	Increased oil content by 14%.	[47]
	Cotton	OE of four individual ACCase subunits	Increased oil content by ~17 to 20%.	[48]
*MCMT*	*Arabidopsis thaliana*	Transgenic line expressing antisense *MCMT*	Impaired growth and cell division.	[49]
		OE of *MCMT*	Increased total fatty acids by 15 to 20%.	
*KAS*	*Arabidopsis thaliana*	T-DNA insertion *KASI* mutant	Reduced total fatty acids by ~34%.	[50]
*HAD*	Rice	Chemical-induced *ZEBRA LEAF16* (encodes for HAD) mutant.	Reduced total fatty acids by ~10%.	[51]
*FAD*	Cotton seeds	Transgenic lines expressing non-functional rapeseed *FAD2*	Reduced seed oil content by ~10%.	[52]
*TE*	*Arabidopsis thaliana*	T-DNA insertion *TE* mutant	Reduced total fatty acids by ~10 to 30%. Reduced TG content by ~50%.	[53]
	Soybean	OE of *TE*	Increased seed oil content by ~20%.	[54]
*LACS*	*Arabidopsis thaliana*	T-DNA insertion *LACS1*, *LACS8* and *LACS9* mutant	Reduced total fatty acids by ~12%.	[55]
	Rapeseed	Transgenic lines with *LACS2* OE or RNAi expression	OE: Increased lipid content by ~3%. KD: Reduced by ~2%.	[56]
	Sunflower seeds	Transgenic lines expressing *Arabidopsis LACS4* and *LACS9* double mutant	Reduced TG content by ~27%.	[57]
*GPAT*	*Arabidopsis thaliana*	Transformation with plastidial safflower *GPAT*	Increased seed oil content by 10 to 21%.	[58]
	*Arabidopsis thaliana*	Seed-specific miRNA *GPAT9* knockdown (KD)	Reduced total fatty acids by ~10 to 30%.	[59]
	Peanut	*GPAT9* OE and KD	OE and KD significantly increased and reduced oil content, respectively.	[60]
	*Arabidopsis thaliana*	OE of rapeseed *GPAT9*	Increased seed oil content by ~10%.	[61]
*LPAAT*	Rapeseed	OE, KD via RNAi and KO via CRISPR-Cas9 of rapeseed *LPAAT2* or *LPAAT5*	OE: Increased seed oil content by ~13 to 16%. KD and KO: Reduced seed oil content, increased sugar and protein by ~15%.	[62]
	*Arabidopsis thaliana*	Transgenic lines overexpressing peanut *LPAAT2*	Increased total fatty acids by ~25%.	[63]
	Rapeseed	OE of *Trapaeolum majus LPAAT*	Increased TG accumulation by up to ~30%.	[64]
	*Arabidopsis thaliana*	OE of rapeseed *LPAAT*	Increased total fatty acids by ~13%.	[65]
*PAH*	*Arabidopsis thaliana*	Transgenic lines expressing the *PAH* RNAi silencing vector	Reduced seed oil content by ~20%.	[66]
*PDCT*	*Camelina sativa*	Transgenic lines overexpressing the *PDCT* gene	Increased seed oil yield by 32 to 76%. FA incorporation into TG and DG, but reduced for PC.	[67]
*PDAT*	Rapeseed	Transgenic lines overexpressing the *PDAT* gene	Reduced seed TG content by ~10 to 20%.	[68]
	*Brassica napus*	Transgenic lines overexpressing the *Sapium sebiferum PDAT1* gene	Increased oil content by ~10%.	[69]
*DGAT*	*Arabidopsis thaliana*	T-DNA KO of *DGAT* OE of *DGAT* cDNA in wild-type lines	KO: Reduced seed oil content by ~5%. KD: Increased seed oil content by ~5–10%.	[70]
	*Arabidopsis thaliana*	*PDAT* RNAi expression in *DGAT1* mutant lines	~20% fatty acid content reduced in *DGAT1* mutant lines. ~80% fatty acid content reduced in *DGAT1* mutant with *PDAT* RNAi.	[71]
	Soybean	Transgenic lines overexpressing *DGAT2*	Increased seed oil content by ~10%.	[72]
	Soybean	Transgenic lines overexpressing peanut *DGAT3*	Increased total fatty acids by ~10%.	[73]
	*Camelina sativa*	Transgenic lines with *DGAT1* silencing and/or *PDAT* OE	*DGAT1* KD reduced the incorporation of radioactive ^14^C into TG. *DGAT1* KD + *PDAT* OE increased the incorporation of radioactive ^14^C into TG and PC, but reduced that into DG.	[74]
	*Camelina sativa*	Transgenic lines with *DGAT1* OE	Increased total fatty acids by ~24%.	[75]
*Oleosin*	Rice	Transgenic lines with embryo-specific soybean *OLEOSIN* OE	Increased seed oil content by ~40%. Increased number of oil bodies with a smaller diameter.	[76]
	Cottonseeds	Transgenic lines with *OLEOSIN* OE	Increased total fatty acid content by ~10 to 20%.	[77]
	*Arabidopsis thaliana*	Transgenic *Arabidopsis oleosin*-deficient lines with sorghum *OLEOSIN* OE	Increased seed oil content by ~30% compared to wild-type and *Arabidopsis oleosin*-deficient lines.	[78]
	Soybean	Transgenic lines with *OLEOSIN* OE	Increased seed oil content by ~10%. Increased number of oil bodies with a smaller diameter.	[79]
	Rice	Transgenic lines with *OLEOSIN* RNAi expression	Reduced TG levels in purified oil bodies by 50%.	[80]

### 3.2. Genomic and Transcriptomic Identification of Targets for Oat Oil Accumulation

In the context of oats, genomics tools, such as genome-wide association studies (GWAS) and quantitative trait loci (QTL) mapping, allowed for the identification of genetic variants linked to variation in lipid content and composition. Using recombinant inbred line populations, *Kanota* × *Ogle* and *Kanota* × *Marion*, a significant QTL affecting oil content was mapped to linkage group 11 [81]. This QTL explained up to 48% of the phenotypic variance in groat oil content within the oat population. Intriguingly, the *ACCase* gene was closely linked to this QTL, suggesting that variations in this gene are central in determining oil levels. Additional loci have been identified that contribute to oil content, but the *ACCase* locus remains the most influential for this trait [81]. Later, more colocalising QTLs have been mapped for groat oil content and fatty acid composition by multiple research groups, which are linked with *ACCase* and *SAD*, respectively [82,83,84,85].

Temporal expression of lipid synthesis-related RNA transcripts in cv. Freja (medium oil) and cv. Matilda (high-oil) varieties were tracked during grain development [86]. Several fatty acid synthesis genes, such as *ACCase*, *KAR*, *ENR*, and *KAS*, exhibited high expression levels at 5 days after pollination (DAP) but subsequently underwent down-regulation, resulting in low expression levels after ~15 DAP. On the other hand, genes involved in later TG assembly processes showed trends opposite to FAS or remained unchanged throughout development [86]. Similar results on the temporal expression patterns of these genes were observed in a more recent study comparing 22 oat lines [87]. While oil is mostly localised in the embryo for many cereal grains, oats uniquely accumulate large quantities of storage oil in the endosperm, particularly in the aleurone layers [88,89,90]. This may be attributed to the region-specific expression of lipid synthesis enzymes. Although the transcripts of *WRI1* homologues were found to be enriched in the oat endosperm, the expressions of *WRI1* downstream target genes did not reflect the high-oil characteristics of the oat endosperm when compared to those in the embryo [91]. Collectively, this highlights the need to consider regulation at the protein and lipid levels. Mass spectrometry (MS)-based omics, particularly proteomics and lipidomics, offer powerful tools to address this knowledge gap and provide a closer representation of the grain oil phenotype.

## 4. Investigation of Oat Oil Regulation Using Mass Spectrometry-Based Omics Approaches

### 4.1. General Principles of MS

Mass spectrometry (MS) is a technique utilised for the detection and quantification of ions based on their mass-to-charge (*m*/*z*) ratio. It comprises three main components: an ion source, a mass analyser, and a detector [92]. The ion source plays a crucial role in converting biological molecules into gas-phase ions through electrospray ionisation (ESI) or matrix-assisted laser desorption and ionisation (MALDI). ESI generates ions from liquid samples, while MALDI ionises biomolecules embedded in a matrix using a laser [93]. These ions are then separated based on their differences in *m*/*z* ratio in a mass analyser, such as the quadrapole, time-of-flight (TOF), or Orbitrap. TOF and Orbitrap mass analysers are widely used in omics studies, including proteomics, metabolomics, and lipidomics. In TOF analysers, ions are accelerated through an electric field into a field-free drift tube, where they travel at different velocities depending on their *m*/*z* ratio [92]. In contrast, Orbitrap analysers operate as ion traps, where the ions oscillate around an electrode, and their oscillation frequencies are measured to determine their *m*/*z* ratio [94]. Ion mobility spectrometry (IMS) is often coupled with MS to further separate ions with similar *m*/*z* ratios. In the gas phase, IMS measures an ion’s collision cross-section (CCS) value, which reflects its structural conformation. This provides an additional dimension for the identification of structural isomers that are unable to be distinguished by mass alone [95,96]. Subsequently, the separated ions are detected using an electron multiplier. The results are then displayed as a mass spectrum, illustrating the signal intensity of detected ions in relation to their *m*/*z* ratio [92].

**Figure 3:**
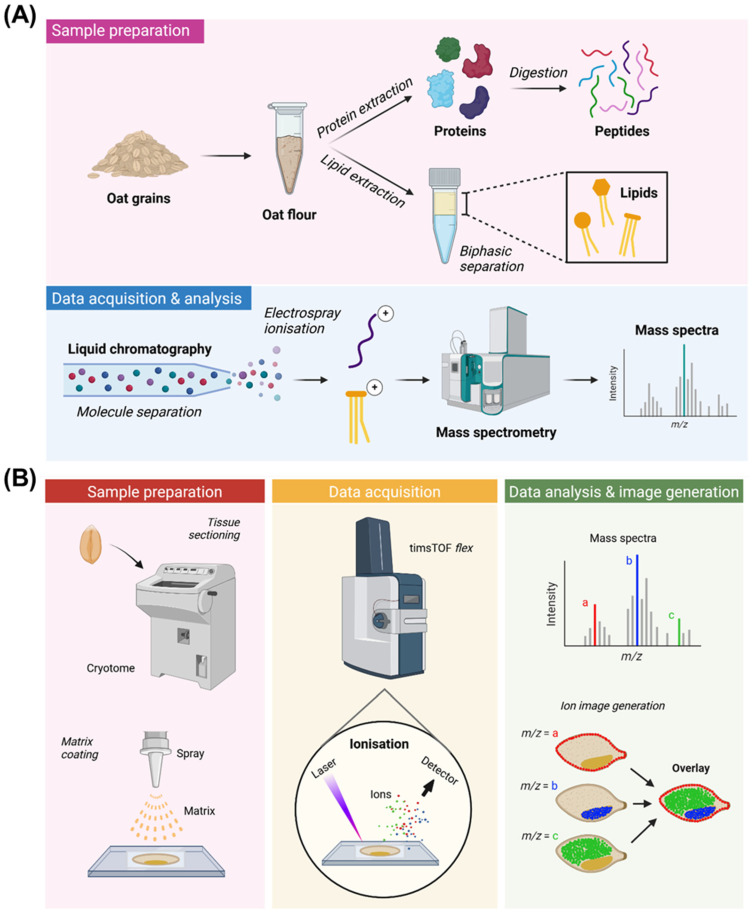
Schematic representing LC-MS/MS and MALDI-MSI workflows using oat grains as an example. (**A**) For LC-MS/MS analysis, proteins or lipids are first extracted from homogenised oat grains. Proteins are digested into peptides prior to LC-MS/MS analysis. Peptides or lipids are separated by LC and subsequently ionised for MS analysis. (**B**) Sample preparation steps for MALDI-MSI include tissue sectioning and matrix coating. The desorption and ionisation of analytes embedded in the matrix is induced by a laser. The resulting ions are accelerated and directed towards a detector. Peaks corresponding to specific ions of interest (based on their *m*/*z*) are selected from the mass spectra for ion image generation. Created using BioRender.com.

A standard LC-MS/MS proteomics workflow typically employs a bottom-up approach, where proteins are first extracted from samples and digested into smaller peptides using trypsin for liquid chromatography (LC) separation (Figure 3A). Peptides are resolved based on their affinities for the stationary phase in a column, allowing them to be eluted at different times. This reduces sample complexity and enables the detection of low-abundant peptides. The LC-separated analytes are then ionised using ESI for MS analysis, and the mass spectrum of the protonated peptide precursors is recorded (MS1 spectrum). These precursor ions are subjected to further fragmentation, commonly by collision-induced dissociation (CID), in tandem MS, resulting in an MS/MS spectrum [97]. There are two main acquisition schemes for bottom-up proteomics—Data-dependent acquisition (DDA) and data-independent acquisition (DIA). DDA selects the most abundant precursor ions based on a set threshold for fragmentation, while in DIA, all ions within predefined *m*/*z* windows across the entire MS1 range are simultaneously fragmented, thus providing a more comprehensive coverage that includes low-abundant ions [98]. The resulting mass spectra are then used for matching against protein databases for annotations. With a similar approach, lipidomics uses a top-down method, as it involves the direct analysis of extracted intact lipids without prior breakdown or digestion (Figure 3A).

The evolution of mass spectrometry imaging (MSI) from conventional MS enables the visualisation of molecular distributions directly from tissue sections of biological samples [99]. MALDI is the most popular ionisation source for MSI, as it can detect a broad spectrum of molecules, including intact proteins, peptides, lipids, glycans, and small molecules. Typically, tissue samples are first embedded in a medium of compatible hardness, cut into thin sections, and mounted onto indium tin oxide (ITO) slides (Figure 3B). A matrix that strongly absorbs UV light is then deposited onto the samples through spraying or sublimation, forming crystals with the analytes on the tissue sample. When irradiated by a short laser pulse, the matrix becomes vibrationally excited and promotes the desorption and ionisation of analytes for MS analysis. MSI captures mass spectra at discrete points, or pixels, to generate spatially resolved molecular information across the sample (Figure 3B). One limitation of MALDI-MSI is its reliance on the MS1 scan, and hence requires follow-up analysis for more confident molecular annotations, either via LC-MS/MS or in situ MS/MS fragmentation of peaks of interest. However, recent advancements in MS instrumentations have enabled the parallel acquisition of MS/MS data, thus improving identification confidence while reducing analysis time [100,101]. Nevertheless, MSI provides valuable spatial context in omics analyses, a feature that is lost in LC-MS/MS due to sample homogenisation.

### 4.2. Proteomic and Lipidomic Interrogation of Oat Oil Synthesis

Oat proteomics previously suffered from the reliance on pan-cereal protein databases, resulting in poor annotations and inaccurate representation of the oat proteome [102,103]. The recent release of a high-quality, fully annotated oat reference genome enables the generation of an oat-specific protein database with translated peptide sequences [21]. Despite this advancement, the functional roles of many enzymes involved in FAS and TG assembly remain poorly characterised in an oat context. Our recent study demonstrated the potential of mass spectrometry-based omics to identify molecular markers associated with oat oil content. In a pilot investigation of five oat varieties, strong correlations between oil content and the abundance of several FAS enzymes, such as ACCase, ENR, and HAD, were observed [104]. However, functional validation of candidate enzymes identified through omics analyses remains essential to validate their contribution to oat oil synthesis.

Lipidomics is a subfield of metabolomics, which refers to the profiling of the lipidome. Variations in lipid species can point to specific enzymatic bottlenecks or regulatory points within the TG assembly pathway. As the formation of TG involves acyl modification of multiple lipid classes, lipidomics can help elucidate the oat TG assembly pathway at the metabolite level. This can be useful in the study of oat lipid synthesis; previous evidence indicated lipid remodelling mechanisms that facilitate phospholipid turnover into TG in high-oil oat genotypes. Heavy carbon label assay indicated a higher efficiency of fatty acid transfer from polar lipids to TG in the high-oil oat, suggesting that the turnover rate of polar lipids may influence the degree of oat oil accumulation [105]. This was further supported by a recent study exploring the oat lipidome of high- and low-oil varieties using developing grains at 9, 17, and 25 days after anthesis, as the authors noted higher levels of lysophospholipids (an indicator of greater phospholipid turnover) in the high-oil oat [106]. However, the study focused only on phospholipids and galactolipids, such as monogalactosyldiacylglycerol (MGDG) and digalactosyldiacylglycerol (DGDG); hence, it remains unknown whether the decrease in phospholipid levels corresponds to increased TG levels during oat grain development.

**Figure 4:**
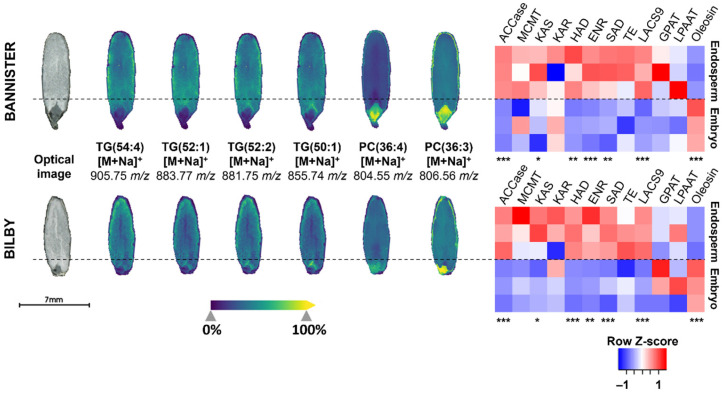
Proteomic and spatial lipidomic analyses of different oat compartments. (**Left**): Ion distribution images of different triacylglycerol (TG) and phosphatidylcholine (PC) adducts detected via MALDI mass spectrometry imaging across tissue sections from two oat varieties (cv. Bannister and cv. Bilby). Signal intensities ranged from low (dark blue) to high (yellow). (**Right**): Heatmap comparing abundances of proteins involved in the FAS pathway (in triplicate) identified in endosperm and embryo fractions (distinguished with dotted lines) via proteomics. Dashed lines indicate the boundary between endosperm and embryo fractions. Adapted (from Figure 6C) from Lau et al. [104] with permission from Elsevier. *, *p* < 0.05; **, *p* < 0.01; ***, *p* < 0.005. ACCase, acetyl-CoA carboxylase; ENR, enoyl-CoA reductase; GPAT, glycerol-3-phosphate acyltransferase; HAD, 3-hydroxyacyl-ACP dehydratase; KAR, 3-ketoacyl-ACP reductase; KAS, ketoacyl-ACP synthase; LACS, long-chain acyl-CoA synthetase; LPAAT, lysophosphatidic acid acyltransferase; MCMT, malonyl-CoA:ACP transacylase; PC, phosphatidylcholine; SAD, stearoyl-ACP desaturase; TG, triacylglycerol; TE, fatty acid thioesterase.

**Figure 5:**
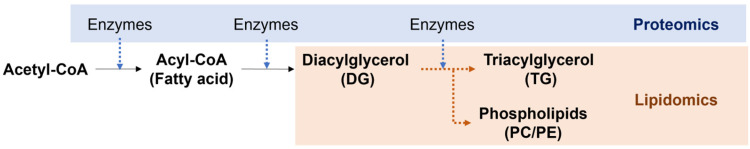
Complementary proteomics and lipidomics to unravel oat lipid biochemistry. Proteomics quantifies the abundance of enzymes governing lipid biosynthesis, while lipidomics characterises the resulting lipid species and their relative levels.

Using a spatial lipidomics approach, it was observed that oil is predominantly deposited in both the central and outermost layers of the oat endosperm. This technique enabled spatial resolution of TG and PC across oat grain sections, revealing distinct accumulation patterns for these lipid classes (Figure 4) [104,107]. TG adduct signals were mainly localised to the endosperm and aleurone layers, consistent with previous observations using nuclear magnetic resonance imaging [86,104]. Proteomic analysis of endosperm fractions revealed significantly higher abundance of FAS enzymes as compared to the embryo (Figure 4), highlighting potential compartment and cell-type specific regulation of lipid synthesis [104]. On the other hand, PC signals were distributed more heterogeneously across oat sections, with higher intensities in the embryo. Interestingly, in lower oil varieties, higher signal intensities of PC adducts were detected in the endosperm [104]. This suggests a lipid remodelling mechanism in high-oil oats, where PC is more efficiently converted into TG, especially within endosperm tissues. In addition, high-oil oats were found to possess higher saturation indices [104,106,108]. It was hypothesised that this phenomenon could arise from the high turnover of phospholipids, which shortens the exposure time of fatty acyl chains to desaturase enzymes. Alternatively, this could be caused by the differential expressions of fatty acid desaturases. In this scenario, a complementary proteomics and lipidomics approach may help to trace the stepwise conversion of fatty acids into TGs through key FAS and TG synthesis enzymes, contributing to a greater understanding of oat lipid biochemistry (Figure 5). Furthermore, lipidomics may serve as an indirect indicator of pathway regulation in cases where TG assembly enzymes are difficult to detect (Figure 5).

## 5. Potential Omics-Driven Insights to Bridge Knowledge Gaps in Oat Lipid Synthesis

### 5.1. Investigation of Oat Oil Synthesis at Cell-Type Resolution

While a cereal grain is primarily composed of endosperm and embryo, they exhibit high levels of cellular complexity. For example, the endosperm is made up of aleurone, sub-aleurone, starchy endosperm, endosperm transfer cells, and embryo-surrounding cells, while the embryo includes scutellum, radicle, and plumule [109,110,111]. These cells undergo distinct spatio-temporal processes during grain development [112,113]. Future studies should focus on investigating oat oil accumulation at cell-type resolution during grain development to further unravel molecular mechanisms. Recently, a study incorporated the laser microdissection (LMD) technique to isolate specific cell types (aleurone, subaleurone, endosperm, and endosperm transfer cells) for proteome and metabolome profiling of developing wheat grains [112]. A deeper understanding of specific cell-type roles of FAS and TG assembly enzymes could inform strategies for low-oil oats while balancing TG and phospholipid ratios to preserve membrane integrity essential for seed growth and development.

### 5.2. Carbon Partitioning Between Macronutrients and Involvement of Transcription Factors

In addition to oil synthesis, understanding the reallocation of carbon flux between oil and other storage molecules, such as starch and protein, is also important to understand how modulating oil content may affect oat yield or nutritional qualities. Ekman and colleagues proposed the oat endosperm as a model system for the study of carbon partitioning between oil and starch, due to the unique accumulation of both molecules in the same tissue type [114]. The negative associations between oil and starch levels were noted in different types of plant tissues, including oats [115,116,117,118] also adds to their reasoning. By using a metabolic flux analysis on oats, it was revealed that heavy-labelled carbon was incorporated more efficiently into the lipid fraction in a high-oil variety, while in a lower oil variety, a greater proportion was accumulated in the starch and protein fractions [114].

Proteomic profiling of developing oat seeds revealed that the low-oil variety exhibited reduced activity in photosynthetic and glycolytic pathways, but elevated expression of starch biosynthesis enzymes, suggesting that oil composition is influenced by the direction of metabolic flux during grain development [108,119,120]. This differential regulation during grain development suggests that transcription factors may be involved in coordinating these processes, particularly *WRI1*. Transcription factors regulate the transcription of genetic information into RNA by binding to specific DNA sequences, and *WRI1* targets sequences upstream of glycolytic and FAS enzymes, rendering regulatory control over these pathways [121,122,123,124]. The potential involvement of *WRI1* is further supported by its high expression in the oat endosperm, but not in the case of wheat, which is known to accumulate oil in the embryo [91]. However, overexpression of *WRI1* in wheat, maize, and rice stimulated oil accumulation in these cereals [45,125,126,127]. In some cases, this occurred at the expense of starch [45,126]. With existing evidence on indirect regulatory functions of WRI1 in regulating starch synthesis genes [45,128], this transcription factor presents as an interesting target for further studies. However, WRI1 detection was not achievable in previous studies, likely due to its low abundance. WRI1 regulation can be studied at the RNA transcript level, which has been widely performed [129], though it may not necessarily correlate with protein expression. We suggest introducing peptide fractionation steps prior to LC-MS/MS analysis to reduce sample complexity and increase the proteome coverage of less abundant proteins [130]. In addition, targeted LC-MS/MS methods could also be used to achieve higher sensitivity. As a complementary approach to proteomics, metabolome profiling of organic acids, sugars, and amino acids may also be more helpful to understand carbon partitioning mechanisms between lipid, starch, and protein.

Apart from carbohydrates, oil and protein content were also shown to be negatively correlated in seed crops such as soybean [131,132,133,134], *Brassica napus* [135], *Arabidopsis thaliana* [136] and sunflower [137]. Comparisons between the proteomes of high- and low-oil oat varieties suggested higher protein synthesising capacities in low-oil oats [104]. However, the mechanisms of carbon reallocation from lipid to protein remain poorly understood. To strengthen these observations and uncover broader trends, future studies should include a larger and more diverse set of oat genotypes.

**Figure 6:**
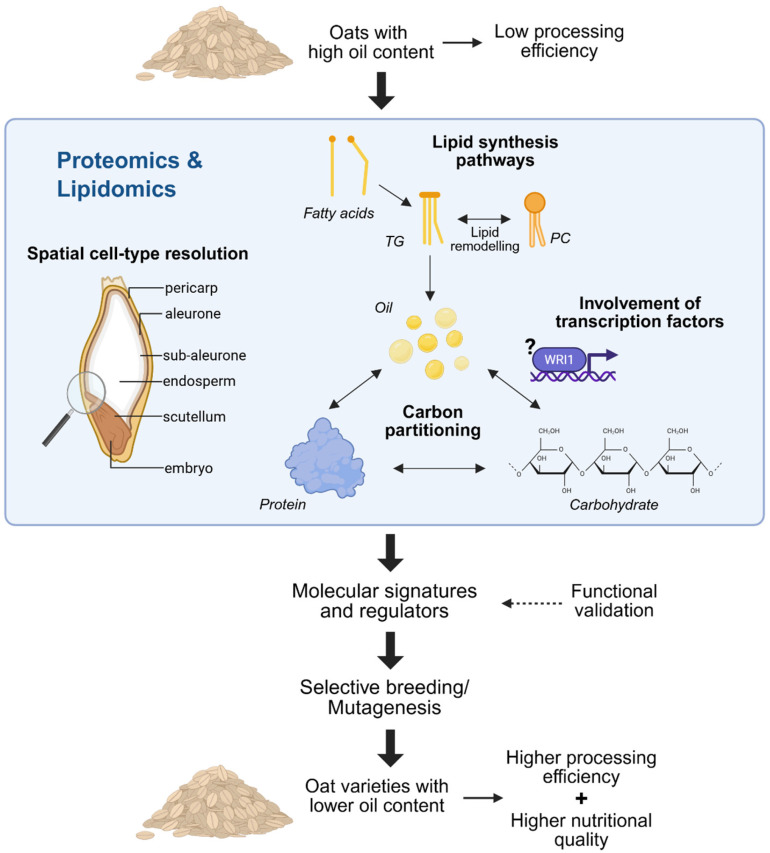
Leveraging mass spectrometry-based omics to unravel lipid regulation for functional crop improvement. Overview of how proteomics and lipidomics can reveal molecular signatures and regulatory candidates for functional validation and their application in breeding programmes for crop quality improvement.

These studies have highlighted the potential of proteomics and lipidomics not only to elucidate the mechanisms underlying oil biosynthesis but also to provide valuable insights into other metabolic pathways, such as protein and starch synthesis, that contribute to overall grain composition and quality (Figure 6).

## 6. Conclusions

Oats are a cereal crop with a unique combination of bioactive compounds that is often overlooked. With the recent trajectory of health-conscious consumers, oats are starting to garner attention as a nutritious plant-based protein alternative or functional ingredient. However, the high oil content of oats restricts industrial processing to extract these nutrients, presenting a significant challenge to diversifying oats for innovative food applications. While developing low-oil oat varieties may be a promising strategy to overcome these limitations, the identification of possible candidates in modulating oil synthesis is essential to achieve this goal. Currently, in an oat context, there is still a lack of molecular knowledge in the interplay between lipid synthesis and other macromolecules like proteins, which may affect overall seed quality. Addressing these gaps is critical for optimising genetic manipulation efforts and ensuring that reductions in oil content do not compromise the nutritional or agronomic value of oats. It is important to consider multiple molecular layers, including genomics and transcriptomics for gene-level insights, alongside proteomics and lipidomics, to achieve a comprehensive understanding of oat oil regulation. With the growing application of proteomics and lipidomics in oat research, we anticipate the identification of additional key regulators of oat oil synthesis in the near future for functional validation. Future studies integrating quantitative assessments of processing efficiency with omics data will provide a valuable framework to link candidate targets to optimal oil content thresholds, ultimately enabling the development of varieties that balance high nutritional value with industrial feasibility.

## Data Availability

No new data were created or analyzed in this study. Data sharing is not applicable to this article.
